# Research protocol: Management of obesity in patients with low health literacy in primary health care

**DOI:** 10.1186/s40608-015-0036-6

**Published:** 2015-02-15

**Authors:** Nighat Faruqi, Nigel Stocks, Catherine Spooner, Nouhad el Haddad, Mark F Harris

**Affiliations:** Centre for Obesity Management and Prevention Research Excellence in Primary Health Care, UNSW Australia, Sydney, Australia; Centre for Primary Health Care and Equity, UNSW Australia, Sydney, Australia; Discipline of General Practice, University of Adelaide, Adelaide, Australia

**Keywords:** Obesity, Primary care nurses, Prevention navigation, Health literacy, Weight management

## Abstract

**Background:**

Socioeconomically disadvantaged adults are both more likely to be obese and have lower levels of health literacy. Our trial evaluates the implementation and effectiveness of primary care nurses acting as prevention navigators to support obese patients with low health literacy to lose weight.

**Methods/Design:**

A pragmatic cluster randomised trial will be conducted. Twenty practices in socioeconomically deprived areas, 10 each in Sydney and Adelaide, will be recruited and randomised to intervention and control groups. Twenty to 40 eligible obese patients aged 40–70 years with a BMI ≥ 30 kg/m^2^ and with low health literacy will be enrolled per practice. The intervention is based on the ‘5As’ of the chronic disease model approach – Assess, Advise, Agree, Assist and Arrange – and the recommendations of the 2013 *Clinical practice guidelines for the management of overweight and obesity in adults*, *adolescents and children in Australia*. In the intervention practices, patients will be invited to attend a health check with the prevention navigator who will assess the patient’s risk and provide brief advice, assistance with goal setting and referral navigation. Provider training and educational meetings will be held. The providers’ attitudes to obesity, confidence in treating obesity and preventive care they provide to obese people with low health literacy will be evaluated through questionnaires and interviews. Patients’ self-assessment of lifestyle risk factors, perception of preventive care received in general practice, health-related quality of life, and health literacy will be assessed in telephone interviews. Patients’ anthropometric measures will be recorded and their health service usage will be determined via linkage to the Australian government-held medical and pharmaceutical data.

**Discussion:**

Our trial will provide evidence for the effectiveness of practice nurses as prevention navigators to support better weight management for obese patients with low health literacy.

**Trial registration:**

This trial is registered with the Australian New Zealand Clinical Trials Registry (ACTRN12614001021662). Date registered 24/09/2014.

## Background

In Australia, obesity is one of the most significant problems facing the healthcare system, with 63% of the population classified as overweight/obese (body mass index [BMI] 25 kg/m^2^ or more) [1]. Obesity is more prevalent among disadvantaged socioeconomic groups than the rest of the population [2].

Obesity is common (27%) among patients presenting in Australian general practice [3]. The ‘5As’ model (Assess, Advise, Agree, Assist and Arrange) provides a framework for preventive care in primary care [4-6]. Providing interventions across the 5As has been advocated in weight management [7-10] and has been shown to increase patient motivation to change their diet behavior [11]. The Australian National Health and Medical Research Council’s *Clinical practice guidelines for the management of overweight and obesity in adults, adolescents and children in Australia* (*the Guidelines*) make recommendations for obesity management in clinical practice across each of the 5As [2]. However, there is evidence that general practitioners (GPs) do not adequately implement these recommendations: a pilot study assessing preventive care for primary care patients with low health literacy found only 15 and 7% of patients in the age group 40–69 years with BMI and waist circumference recorded, respectively [12]. Another study has found low rates of lifestyle advice and referral [13]. These gaps in performance have been attributed to a variety of patient, provider, practice and system factors [14,15].

Health literacy is the capacity to obtain, process, and understand basic health information and services needed to make appropriate health decisions [16]. People with low health literacy are more likely to suffer higher chronic disease morbidity and mortality [17,18] and less likely to receive preventive care [19]. Over 20% of the Australian population has very low health literacy and this is more prevalent among those from disadvantaged socioeconomic backgrounds [20].

Nurses have potential to help adult patients improve health behaviours [21] and to deliver interventions for obesity management in general practice [22]. The feasibility of practice nurses (PNs) in obesity prevention and management has been demonstrated in Australian general practice [23,24]. The objective of our trial is to evaluate the implementation and effectiveness of using PNs in the role of ‘prevention navigators’ to support obese patients with low health literacy to better manage their weight.

### Hypotheses

#### Primary hypotheses

1. PNs acting as prevention navigators in intervention practices will demonstrate greater improvement in their self-reported behaviour and confidence in assessing obese patients with low health literacy and providing advice and referral to them for weight loss compared with PNs in the control group.
2. Recruited patients (obese patients with low health literacy) attending intervention practices will be, at six months, more likely to report having (i) received assessment, advice and referral for weight loss and (ii) attended/used community-based weight loss lifestyle modification programs to which they have been referred to compared with patients in control practices.
3. Recruited patients attending intervention practices will be, at 12 months, more likely to have improved their health literacy related to weight loss than patients in control practices.

### Secondary hypotheses

1. Recruited patients attending intervention practices will be, at 12 months, more likely to have (i) increased intake of dietary portions of fruit and vegetables per day and minutes of moderate and/or vigorous intensity physical activity per week, (ii) reduced consumption of high fat food and hours of sedentary activity per day, and (iii) moderated alcohol consumption compared with patients attending control practices.
2. Recruited patients attending intervention practices will be, at 12 months, more likely to (i) have reduced their baseline weight by 5% or maintained it and (ii) report improvements in their health-related quality of life compared with patients attending control practices.
3. The total cost of health service use of patients in the intervention and control practices will not be different.
4. GPs in intervention practices will demonstrate greater improvement in their self-reported behaviour and confidence in assessing obese patients with low health literacy and providing advice and referral to them for weight loss compared with GPs in the control group.

## Methods/Design

### Research design

A pragmatic cluster-randomised controlled trial will be conducted with randomisation of practices to intervention and control groups. Randomisation will be in permutated blocks and will occur across the two cities. Due to the nature of the intervention, participating GPs and PNs will not be blinded to the intervention.

### Timeline

The intervention will be delivered over a period of 6 months.

### Trial participants

#### General practices

The South Western Sydney and Central Adelaide and Hills Medicare Locals will assist the research team to identify eligible practices. The eligibility criteria include practices being situated in low socioeconomic areas, having electronic medical records that can be audited and with at least one consenting GP and PN each. Practices will be emailed or faxed a synopsis of the trial protocol and invited to participate. Non-responding practices will be contacted by telephone. Once a practice has indicated a willingness to participate, the research team will visit the practice and explain the trial to the interested GPs and PNs. Twenty practices will be recruited, 10 each in Sydney and Adelaide.

#### Patients

The patient eligibility assessment and recruitment will be conducted over a 4 week period. During this period, posters will be displayed in the waiting room informing patients of practice participation in the trial. The patient eligibility assessment will be carried out in 2 steps:

1. Software, specifically developed for this trial, will be installed on the reception computer(s). The software will identify which patients scheduled to visit in each session are potentially eligible to participate. The eligibility criteria include: aged 40–70 years, at least one practice attendance in the previous 12 months, and the GP the patient usually attends being a trial participant. The software will exclude patients who have the following: heart disease, stroke, insulin-treated diabetes, and chronic renal impairment (eGFR < 60 mls/minute/1.73 m^2^). Patients having current treatment with a weight loss medication (orlistat or phentermine) and previous or planned bariatric surgery in the next 12 months will also be excluded.
  For each eligible patient the software will print an enrollment form, which includes four parts: (i) a health literacy screening questionnaire, (ii) an eligibility-determining data form, (iii) a patient consent form, and (iv) a pre-filled clinical data form. The receptionist will hand out the enrollment form together with the patient information sheet and ask the patient to complete the first part (health literacy screening questionnaire) in the waiting room. Patients who have insufficient proficiency in the English language but can complete the screening questionnaire in a language for which a translated questionnaire and patient information sheet are available, will receive the forms translated into their own language.
  The screening questionnaire will contain a slightly modified version of the three questions developed by Chew *et al.* (Table 1) [25]. These have been used in our own pilot study [12] and in other studies with disadvantaged populations [26,27]. A fourth question will assess patients’ language preference for reading medical or health care instructions [28]. To encourage inviting all potentially eligible patients in the screening survey, the reception staff will receive a $200 gift voucher.

**Table 1:** **Health literacy screening questionnaire**

A. How often do you have someone help you read health information materials?	1. Never
	2. Occasionally
	3. Sometimes
	4. Often
	5. Always
B. How often do you have problems learning about your medical condition because of difficulty understanding health information materials?	1. Never
	2. Occasionally
	3. Sometimes
	4. Often
	5. Always
C. How confident are you filling in medical forms by yourself?	1. Extremely
	2. Quite a bit
	3. Somewhat
	4. A little bit
	5. Not at all
D. In which language do you prefer to read your health care information?	English
	Other
2. GPs will score the completed health literacy screening questionnaire, fill in the eligibility-determining data form, and assess the overall patient eligibility. The patient will be considered eligible if the combined score of the three health literacy questions is >10 [27] or the score of the question *“How confident are you filling in medical forms by yourself?”* is ≥3 [25] and the patient has a BMI ≥30 kg/m^2^, is willing to return for a health check (only in intervention practices), and does not have any of the exclusion criteria.

### Intervention description

The intervention has two parts: a clinical intervention and a practice-level intervention.

**Figure 1:**
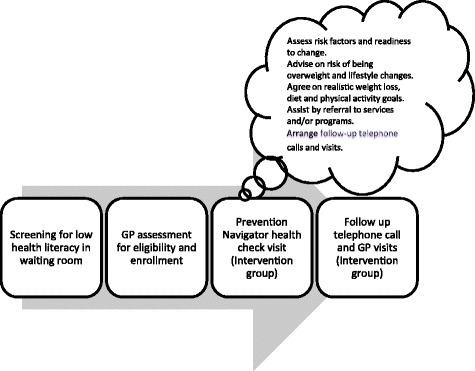
**Patient involvement.**

Clinical intervention: This will be delivered over a 6-month period by GPs and PNs acting as prevention navigators. As recommended by *the Guidelines,* other guidelines and other research, the clinical intervention is based on the 5As framework [2,4,5,7], comprising: (i) Assessment of health literacy, obesity, dietary habits, physical activity levels and readiness to change (ii) Advice on risk of being overweight and lifestyle changes (iii) Agreement on realistic weight loss, diet and physical activity goals (iv) Assisting by referral to services and/or programs and (v) Arranging follow-up telephone calls and visits to review progress. Steps i-iv will be delivered at a health check visit about 2 weeks after the health literacy screening and enrollment of patients in the trial (Figure 1).

Practice-level intervention: This is focused on engaging the practice in implementing the clinical approach stated above with obese patients with low health literacy. In this regard two interactive face-to-face meetings will be held at individual practices. At the first meeting, reports which are based on a baseline clinical record audit will be discussed. The Chief Investigators will use the clinical audit data as a prompt to discuss strategies for improvements in primary cardiovascular disease (CVD) risk factor assessment and management specifically for obese patients based on *the Guidelines*. At another meeting, results from the health literacy screening will be discussed to raise the awareness of the health professionals of the prevalence of low health literacy amongst their patients and the need to pay special attention to these patients.

There will be a 3-hour interactive group training of GPs and PNs in providing weight management to obese patients with low health literacy. This session will include case scenario discussion and role plays. At this training, the intervention group will be provided with a copy of *the Guidelines*.

The other major component of the practice-level intervention is to develop the role of the PNs as prevention navigators. The principal role of a prevention navigator is to work with patients to set goals, provide information and support to choose appropriate referral options (including group programs and telephone coaching), overcome barriers to attendance at this referral and arrange follow up. This is a new potential role for PNs in Australian general practice although there have been some previous examples of the use of community navigators in screening and prevention [29-31].

We will train PNs in their role as prevention navigators and in the conduct of the health check visit. Training will focus on communication with patients with low health literacy and facilitating goal setting, referral navigation and arranging follow up visits.

Educational meetings will also be held for GPs in the control group. However, the educational messages delivered will not be in the same context of obesity management as for the intervention group. For example, at the clinical audit result discussion, emphasis will be on CVD risk factor recording and management. The control group will also be provided with a copy of *the Guidelines*.

### Ethics

Ethics approval has been granted by the UNSW Australia Human Research Ethics Advisory Panel and the University of Adelaide Human Research Ethics Committee. All participants will give written informed consent.

### Data collection

#### Evaluation of general practitioners and practice nurses

Data will be collected at baseline and 12 months to compare intervention and control groups through surveys and interviews and will afford linkage with patient-level data. PNs will be additionally interviewed about prevention navigation at 12 months only.

- GP and PN Surveys: A validated self-completed questionnaire [32] which was modified for our previous research [12] has been further adapted for this trial. The questions relate to the assessment and management of CVD risk factors and GP and PN confidence in managing obese patients with low health literacy. We have included questions to assess GP and PN attitudes, beliefs and practices regarding obesity management [33] and their knowledge about obesity assessment.
- GP and PN Qualitative Interviews: GPs and PNs will be invited to participate in qualitative interviews. The interviews will assess their provision of preventive care to obese patients with low health literacy including the perceived facilitators and barriers and educational needs for effective management of obesity. In addition, we will explore how patient ethnicity affects their weight management.

Additional questions will explore the role of PNs in helping patients navigate community-based lifestyle referral services for weight loss and the perceived facilitators and challenges in doing so. Barriers that patients face in accessing referral services will also be discussed.

#### Evaluation of patients

All patients will be surveyed at baseline, 6 and 12 months. In the intervention group, a subsample of Arabic and English-speaking patients will also be interviewed at baseline and 12 months. Data on anthropometric measures will be collected at baseline and 12 months.

- Patient Surveys: A questionnaire will be administered by telephone in English or in the language in which patients completed the health literacy screening questionnaire. The survey used at baseline and 12 months includes the following five sections:
  i. Assessment of lifestyle-related risk factors and management by primary healthcare providers and patient’s stage of change.
  ii. The 12-item short form – the SF-12® Health Survey – with a 4-week recall to measure eight health-related quality of life domains: physical functioning; role-physical; bodily pain; general health; vitality; social functioning; role-emotional; and mental health [34].
  iii. Health Literacy Questionnaire to assess patients’ general health literacy needs and challenges. This questionnaire covers nine conceptually distinct areas of health literacy that capture a wide range of the lived experiences of people attempting to engage in understanding, accessing and using health information and health services [35]. The domains are: feeling understood and supported by healthcare providers; having sufficient information to manage my health; actively managing my health; social support for health; appraisal of health information; ability to actively engage with healthcare providers; navigating the healthcare system; ability to find good quality health information; and understanding health information well enough to know what to do.
  iv. Impact of Weight on Quality of Life-Lite (IWQOL-Lite) [36], a validated, 31-item, self-report tool, to measure obesity-specific quality of life. In addition to a total score, there are scores on five domains: physical function; self-esteem; sexual life; public distress; and work.
  v. Demographic questions such as age, sex, and country of birth.
    At 6 months, patients will be administered a shortened version of the survey covering only assessment and management of lifestyle-related risk factors and stage of change.
- Patient Interviews: A subsample of Arabic and English-speaking patients, in the intervention group, will be qualitatively interviewed over telephone. The aim for this interview is to explore ethnicity-related factors which influence health literacy for weight management.
- Anthropometric Measures: At baseline, at the time of patient enrollment, GPs will check patient weight and height and calculate BMI. This information will be recorded on the enrollment form. At 12 months, the research team will send invitation letters to patients to attend their practice for final data collection. After two weeks, reminders will be sent to patients who have not responded to the initial invitation. At this visit, data will be collected on weight, BMI and current use of medicines which could impact body weight.

Besides the data collected through surveys, interviews and anthropometry, we will also collect data from clinical records, health literacy screening and health checks.

- Clinical Record Audits
  Audits of clinical records will be conducted at the baseline and 12 months in all practices. Electronic records of patients, aged 40–70 years, under the care of participating GPs will be audited for CVD risk factors with a tool that allows unidentifiable data extraction [37]. The CVD risk factors include: weight, height, BMI, waist circumference, smoking status, alcohol consumption, blood pressure, lipids and 5-year absolute CV event risk. All GPs will be sent reports based on the clinical record audit results. The reports for the intervention and control groups will, however, cover different aspects of the clinical audit results.
- Health Literacy Screening
  Health literacy screening will take place in all the participating practices. Patients, 40–70 years old, will be screened over a 4-week period using a questionnaire, as described earlier. The self-administered questionnaire is scored on a 5-point 1–5 scale [27]. Scores for the three screening questions will be dichotomised into adequate (3–10) or low health literacy (>10) [27]. Patients whose score of the question *‘How confident are you filling in medical forms by yourself?’* is ≥3, will also be categorised as having low health literacy [25].
  The screening will identify patients who have low health literacy and who will be potentially eligible for recruitment.
- Health Checks
  Health checks will take place in intervention practices only. At the health check visit, prevention navigators will collect data on risk assessment which will include weight, BMI, waist circumference, dietary habits and physical activity. Patients will be assessed for readiness to change their weight and barriers to changing diet and physical activity will be determined. Weight, diet and physical activity goals will be set and recorded. The assessment data will help the prevention navigators to tailor their advice to the patients’ need and to help patients decide about their referral to community-based lifestyle modification programs/services. Data on goal setting will be used to determine patient’s progress against the goals at follow-up visits.

### Outcomes

The proposed primary outcome measures are:

1. PNs’ self-reported behaviour and confidence in assessing obese patients with low health literacy and providing advice and referral to community-based lifestyle modification programs for weight loss.
2. Patients’ report, in past six months, of receiving assessment, advice and referral for weight loss and attending/using community-based weight loss lifestyle modification programs referred to.
3. Patient health literacy related to weight loss.

The proposed secondary outcome measures are:

1. Patient self-reported (i) intake of dietary portions of fruit and vegetables per day, (ii) use of high fat food per day, (iii) hours of sedentary activity per day, (iv) consumption of alcohol, and (v) minutes of moderate and/or vigorous intensity physical activity per week.
2. Measured weight.
3. Health-related quality of life.
4. The total cost of health service use.

### Sample size and data analysis

Estimates for sample size based on intra-cluster correlation coefficients for clustering, prevalence, variance and effect sizes from our previous research are in Table 2. Sample size estimates are based on a two-sided test of significance at α = 0.05. β = 0.8 and 20% loss to follow up and effect sizes and standard deviations based on our previous research [38,39].

**Table 2:** **Estimates of sample size for primary and secondary outcomes**

**Outcome**	**Intra-cluster correlation coefficient**	**Design effect (30 patients per practice)**	**Effect size/different proportions**	**Sample size estimate for each group**
Advice on diet	0.051	2.48	25%	170
Advice on physical activity	0.035	2.02	25%	140
Referral/attendance diet	0.026	1.75	25%	115
Referral/attendance physical activity	0.011	1.32	25%	80
Mean health literacy score	0.014	1.41	0.4	140
Mean diet score	0.001	1.03	0.4	115
Mean physical activity score	0.018	1.52	0.4	150
Mean BMI	0.038	2.10	0.5	135

We will examine change in the outcomes between intervention and control practices, after adjusting for baseline differences. We will analyse patient variables for within and between practice differences using multilevel linear and logistic regression techniques that adjust for clustering by practice and multiple imputation to adjust for missing values. Analyses will be on an intention to treat basis.

### Project management

The trial will be led by a project management committee which comprises the investigators, the project coordinator and field researchers and meets bimonthly. In each city a working group manages the field operations in collaboration with their own Medicare Local.

### Trial status

The trial is registered with the Australian Clinical Trials Registry ACTRN12614001021662.

The trial is underway with baseline data collection completed and the intervention commenced.

## Discussion

The BMWGP trial aims to evaluate the implementation and effectiveness of PNs acting as prevention navigators supporting obese patients with low health literacy to better manage their weight. This has the potential to address both the infrequency of referral for obese patients in Australian primary care and the barriers to preventive care experienced by patients with low health literacy through an intervention at both the clinical and practice levels. It also has significant implications for the role of PNs and other health professionals in preventive care in general practice and will inform current discussion of how this role needs to develop in the future [40,41].
